# Cortical morphometry and structural connectivity relate to executive function and estradiol level in healthy adolescents

**DOI:** 10.1002/brb3.1413

**Published:** 2019-09-30

**Authors:** Teodora Stoica, Lindsay Kathleen Knight, Farah Naaz, Melina Ramic, Brendan E. Depue

**Affiliations:** ^1^ Interdisciplinary Program in Translational Neuroscience University of Louisville Louisville KY USA; ^2^ Department of Psychiatry and Behavioral Sciences Johns Hopkins University School of Medicine Baltimore MD USA; ^3^ Department of Psychiatry University of Miami Coral Gables FL USA; ^4^ Department of Psychological and Brain Sciences University of Louisville Louisville KY USA; ^5^ Department of Anatomical Sciences and Neurobiology University of Louisville Louisville KY USA

**Keywords:** adolescent, anisotropy, estradiol, executive function, gray matter, white matter

## Abstract

**Introduction:**

Emotional and behavioral control is necessary self‐regulatory processes to maintain stable goal‐driven behavior. Studies indicate that variance in these executive function (EF) processes is related to morphological features of the brain and white matter (WM) differences. Furthermore, sex hormone level may modulate circuits in the brain important for cognitive function.

**Methods:**

We aimed to investigate the structural neural correlates of EF behavior in gray matter (GM) and WM while taking into account estradiol level, in an adolescent population. The present study obtained neuroimaging behavioral and physiological data from the National Institute of Health's Pediatric Database (NIHPD). We analyzed the relationship between cortical morphometry and structural connectivity (*N* = 55), using a parent‐administered behavioral monitoring instrument (Behavior Rating Inventory of Executive Function—BRIEF), estradiol level, as well as their interaction.

**Results:**

Executive function behavior and estradiol level related to bidirectional associations with cortical morphometry in the right posterior dorsolateral prefrontal cortex (pDLPFC) and primary motor cortex (PMC), as well as fractional anisotropy (FA) in the forceps major and minor. Lastly, the interaction of EF behavior and estradiol level related to decreased volume in the right PMC and was linked to altered FA in the right inferior fronto‐occipital fasciculus (iFOF).

**Conclusions:**

The study provides evidence that the relationship between EF behavior and estradiol level related to bidirectional GM and WM differences, implying estradiol level has an influence on the putative structural regions underlying EF behavior. The findings represent a crucial link between EF behavior and hormonal influence on brain structure in adolescence.

## Graphical abstract

The comprehensive surface‐based morphometry study provides evidence that the relationship between executive function behavior and estradiol level related to bi‐directional grey matter and white matter differences, implying estradiol level has an influence on the putative structural regions underlying executive function behavior. The findings represent a crucial link between executive function behavior and hormonal influence on brain structure in adolescence.

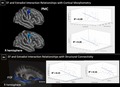

## 1 INTRODUCTION

Adolescence represents a dynamic developmental stage that corresponds with dramatic changes in brain architecture as it remodels itself to sustain the demands of a young adult physiology (Arain et al., 2013; Asato, Terwilliger, Woo, & Luna, 2010; Blakemore & Choudhury, 2006; Paus, 2005). Previous studies consistently demonstrate a nonlinear change in cortical gray matter (GM), while in contrast, white matter (WM) exhibits a steady linear increase during adolescence (Arain et al., 2013; Giedd et al., 1999; Paus, 2005; Sowell, Thompson, Holmes, Jernigan, & Toga, 1999). This monumental neural reorganization driven by gonadal hormone exposure is thought to increase neural efficiency between the prefrontal cortex (PFC) and other posterior cortical structures. These structures subserve executive functions (EF), a set of processes necessary for seamless integration of top–down cortical control which forms the basis of goal‐directed behavior (Blakemore & Choudhury, 2006; Caballero, Granberg, & Tseng, 2016; Giedd et al., 1999; Paus, 2005; Smolker, Depue, Reineberg, Orr, & Banich, 2015; Sowell et al., 1999).

### 1.1 Relationship between executive function and cortical morphometry

Despite EF's critical role in guiding future‐oriented behavior, inconsistencies exist regarding the morphological features that support it during adolescence. While some studies demonstrate that increases in total cortical and PFC GM volume (GMV) relate to higher scores on working memory and response inhibition tests (Kharitonova, Martin, Gabrieli, & Sheridan, 2013; Mahone, Martin, Kates, Hay, & Horska, 2009; Yurgelun‐Todd, 2007); others show that GMV decreases in the PFC relate to increased ability to regulate emotion, better working memory capacity, and higher scores on verbal memory tests (Caballero et al., 2016; Yurgelun‐Todd, 2007). Similarly, higher IQ during adolescence is associated with cortical thinning of left superior orbitofrontal cortex and superior motor area, and higher bilateral hemispheric surface area (SA) (Schnack et al., 2015). Thus, bidirectional morphological results in relation to EF need not be interpreted as contradictory, instead they could possibly reflect the fact that GM maturation follows an inverted‐U shape over development, peaking at different ages depending on the region (Ducharme et al., 2015; Sowell, Thompson, Tessner, & Toga, 2001). Therefore, GM is considered closely related to maturation of a brain region (Crone, 2009; Giedd, 2004), suggesting that controlling for age is paramount when examining cortical GM in adolescent samples.

### 1.2 Relationship between executive function and structural connectivity

In a more consistent pattern than GM maturation during adolescence, studies examining fractional anisotropy (FA; a WM integrity descriptor) during this period indicate relatively linear increases coinciding with improved EF performance. Specifically, increases in FA in the posterior corpus callosum during adolescence are associated with better working memory and IQ scores (Giedd, 2004; Giorgio et al., 2010; Nagy, Westerberg, & Klingberg, 2004). Similarly, research indicates increased FA of fronto‐temporal‐subcortical WM tracts (inferior fronto‐occipital longitudinal fasciculus (iFOF), superior longitudinal fasciculus (SLF), arcuate fasciculus and the corticospinal tract) support enhanced communication between disparate regions of the cortex and reflect increases in top‐down cognitive control of behavior (Asato et al., 2010; Peper, Heuvel, Mandl, Pol, & Honk, 2011). While previous studies show specific changes in GM and FA and indicate relationships with some facets of EF, a comprehensive cortical morphometry and structural connectivity investigation concerning the full range of EF constructs is lacking.

### 1.3 Measuring executive function in children and adolescents

A tool often used to investigate multiple EF constructs and occasionally, their underlying neural substrates, is the Behavior Rating Inventory of Executive Function (BRIEF). This reliable and validated psychological battery is designed to measure EF behavior in children and adolescents (5–18 years) during everyday situations through behavioral observation (Clark, Pritchard, & Woodward, 2010). Initial factor analytic studies of the BRIEF support two robust indices: a Behavioral Regulation Index (BRI)—emphasizing inhibitory and emotional control (EC), and a Metacognition Index (MCI)—emphasizing working memory, planning, and strategic response preparation (Mahone et al., 2009). The sum of the two indices provides a Global Executive Composite (GEC), whereby elevated scores indicate more observed problems with EF behavior. Neurobiologically, research indicates that the BRIEF captures unique variance in predicting PFC development in children and adolescents (Mahone et al. (2009) and provides an economical port of entry to both behavioral regulation and cognitive issues that may in turn relate to cortical morphometry and structural connectivity measurements.

### 1.4 Relationship between executive function, cortical morphometry and structural connectivity

However, findings from the few studies that have investigated cortical morphometry in healthy, typically developing adolescents in relation to EF are inconsistent, likely due to the magnitude of change during adolescence. While some studies demonstrate that increased frontal GMV relates to decreased working memory and emotional control (Faridi et al., 2015; Mahone et al., 2009), others show the inverse pattern: decreased temporal lobe GMV relates to decreased inhibition and emotional control (Faridi et al., 2015). Conversely, relationships between structural connectivity and the BRIEF during this variable period reflect an evident pattern: Reductions in FA relate to decreased EF behavior. A variety of pediatric clinical populations exhibit reduced FA in temporal, frontal, and corpus callosal regions in association with deficits on the GEC (Antshel, Conchelos, Lanzetta, Fremont, & Kates, 2005; Gautam et al., 2015; Wozniak et al., 2007). The sole study linking EF behavior with FA in a healthy adolescent sample investigated the frontal aslant tract (FAT), a newly discovered white matter tract which connects posterior inferior frontal gyrus (IFG) with the pre‐supplementary and supplementary motor areas (pre‐SMA and SMA), regions proposed to underlie inhibition. The study indicates the FAT develops in a protracted manner into late adolescence/early adulthood and that right lateralization of this fiber pathway is significantly associated with decreased EF behavior as measured by the BRIEF (Garic, Broce, Graziano, Mattfeld, & Dick, 2018). Taken together, scant evidence indicates that EF behavior is associated with both GM and FA changes during childhood and adolescence, yet the results are conflicting. Therefore, a comprehensive cortical morphometry and structural connectivity study using the BRIEF to assess EF behavior within a healthy sample of adolescents can help clarify previous findings.

### 1.5 Relationship between executive function, cortical morphometry, structural connectivity and estradiol

During this developmental period of high flux, the hormone estradiol, the predominant estrogen, has been shown to have a significant impact on the structural reorganization of the prefrontal cortex (McCarthy, 2008; Nguyen et al., 2013), a crucial region underlying EF (Yuan & Raz, 2014). The hormone has complex effects in the two genders, however, because estrogen receptor distribution in the prefrontal cortex varies (Cooke, Nanjappa, Ko, Prins, & Hess, 2017; Gillies & McArthur, 2010). Therefore, estradiol may have both similar and different (sometimes opposite effects) due to underlying brain dimorphisms. Nonetheless, this hormone influences cognitive function through complex interactions with dopaminergic and oxytocinergic systems that govern EF (Kuhn et al., 2010; Steinberg, 2005), the description of which is not within the scope of this paper. The complex, menstrual phase‐dependent evidence from studies in adult women points to estradiol level playing both a facilitative and/or hindering role in cognitive function. Some studies report higher levels of circulating estradiol being associated with improved working memory performance (Hampson & Morley, 2013), while others show increased estradiol had a negative impact on general processing speed, working memory performance (Sommer et al., 2018), and slower response times and decreased accuracy on EF tasks that were instead related to progesterone level during the luteal phase (Hidalgo‐Lopez & Pletzer, 2017). Alongside the adult literature, morphometric studies in young adults demonstrate increased circulating levels of estradiol are associated with cortical thinning of the IFG (Witte, Savli, Holik, Kasper, & Lanzenberger, 2010), a region linked to self‐regulation (Aron & Poldrak, 2006; Depue, Curran, & Banich, 2007; Depue, Orr, Smolker, Naaz, & Banich, 2015). Structural connectivity evidence points to elevated estradiol level influencing decreases in FA, which is associated with reduced behavioral control during early pubertal development (Peper, Reus, Heuvel, & Schutter, 2015). Elevated estradiol level in adolescent girls shows a negative relationship with right angular gyrus (AG) and the superior longitudinal fasciculus (SLF) FA, a brain region and a WM tract involved in attention, spatial and social cognition (Herting, Maxwell, Irvine, & Nagel, 2012). However, scant evidence between the relationship between EF behavior, estradiol, and specific brain changes exists.

Therefore, the present study aimed to comprehensively investigate the relationship between EF behavior (as measured by the BRIEF questionnaire) and estradiol level, individually and interactively on cortical morphometry and FA in a healthy adolescent sample. Specifically, the aims were to examine the relation between (a) the BRIEF and estradiol level, (b) the BRIEF, cortical morphometry, and FA, (c) estradiol level, cortical morphometry and FA, and (d) any interaction between the BRIEF and estradiol level with cortical morphometry and FA. In parallel, we hypothesized based on the limited literature findings that (a) EF behavior and estradiol level will be inversely related, (b) decreased EF behavior will relate to decreased GMV of the LPFC and decreased FA of WM tracts subserving EF (iFOF/SLF), (c) increased estradiol level will relate to decreased FA and cortical morphometry of the LPFC, and (d) increased estradiol level combined with decreased EF behavior would subsequently exacerbate these previous findings. This comprehensive study, therefore, investigated how individual differences in EF behavior and estradiol level relate to variation in aspects of cortical morphometry and FA in a healthy, adolescent sample.

## 2 METHODS

### 2.1 Participants

Cross‐sectional data were obtained from the Pediatric MRI Data Repository (Release 4.0) of the NIH MRI Study of Normal Brain Development, a project developed to characterize healthy brain maturation in relation to behavior in a large, multisite study (Evans & Brain Development Cooperative, 2006). This multi‐center project conducted epidemiologically based recruitment of a large, demographically balanced sample across a wide age range, using strict exclusion factors and comprehensive clinical/behavioral measures. A mixed cross‐sectional and longitudinal design was used to create an MRI/clinical/behavioral database from approximately 500 children, aged 7 days to 18 years, to be shared with researchers and the clinical medicine community. Using a uniform acquisition protocol, data were collected at six Pediatric Study Centers and consolidated at a Data Coordinating Center. Enrolled subjects underwent a standardized protocol to characterize neurobehavioral and pubertal status. The data were demographically representative of the U.S. population in terms of variables including gender, race, and socioeconomic status (Waber, Forbes, Almli, Blood, & Cooperative, 2012). Exclusion criteria included but were not limited to IQ < 70, history of medical illness with CNS implications, and any Axis I psychiatric disorder (other than simple or social phobia, adjustment disorder, oppositional defiant disorder, enuresis, encopresis, or nicotine dependency; see Waber et al. (2007) for a complete list of inclusion and exclusion criteria). Participants underwent brain MRIs and extensive neuropsychological testing on up to three occasions at two‐year intervals. For the purposes of this report, a sample of 55 participants (age range 7–18) with cross‐sectional data (1 time point) was selected with structural imaging data (T1), diffusion tensor imaging data (DTI), behavioral (BRIEF), and hormonal data (estradiol) (Table 1). Seven participants were missing estradiol data; therefore, they were not included in subsequent analyses involving estradiol. Collection site was treated as a nuisance factor in all subsequent analyses.

**Table 1:** Descriptive statistics

Variable	*N*	Range	Mean ± *SD*
Age	55	7–18	13.7 ± 3.5

Variable	*N*	Percentage
Gender		
Male	21	38
Female	34	62
Race		
White	45	82
African American	4	7
Others	6	11
Household income		
$25–35,000	3	5
$35–50,000	11	20
$50,001–75,000	16	29
$75,001–100,000	13	24
$100,001–150,000	10	18
Over $150,000	2	4

### 2.2 Behavioral measures

#### 2.2.1 Behavior rating inventory of executive function (BRIEF)

The BRIEF was completed on the same day as the scan by a parent or guardian that had contact with the child within the prior 6 months. The 86‐item questionnaire takes approximately 10 min to administer and can be administered and scored by a research assistant. The test was divided into the Behavioral Regulation Index (BRI) which comprised: Inhibit, Shift, Emotional Control subscales, and the Metacognition Index (MCI) which comprised: Initiate, Working Memory, Plan/Organize, Organization of Materials, and Monitor subscales. A higher score on each of the subscales signified *decreased* EF behavior. Subscales (Table 2) were used for further correlation and regression analyses with cortical, FA, and hormonal measurements, controlling for age and gender. Multiple comparison correction was carried out using the Benjamini & Hochberg, 1995 procedure (Benjamini & Hochberg, 1995), controlling the false discovery rate (FDR) at *p *< .05.

**Table 2:** BRIEF subscale descriptions

Inhibit	Ability to control impulses (inhibitory control) and to stop engaging in a behavior
Shift	Ability to move freely from one activity or situation to another; to tolerate change, to switch or alternate attention
Emotional control	Ability to regulate emotional responses appropriately
Initiate	Ability to begin an activity and to independently generate ideas or problem‐solving strategies
Working memory	Ability to hold information when completing a task, when encoding information, or when generating goals/plans in a sequential manner
Plan/Organize	Ability to anticipate future events; to set goals; to develop steps; to grasp main ideas; to organize and understand the main points in written or verbal presentations
Organization of materials	Ability to put order in work, play, and storage spaces (e.g., desks, lockers, backpacks, and bedrooms)
Monitor	Ability to check work and to assess one's own performance; ability to keep track of the effect of one's own behavior on other people

### 2.3 Physiological measures

#### 2.3.1 Estradiol

**Figure 1:**
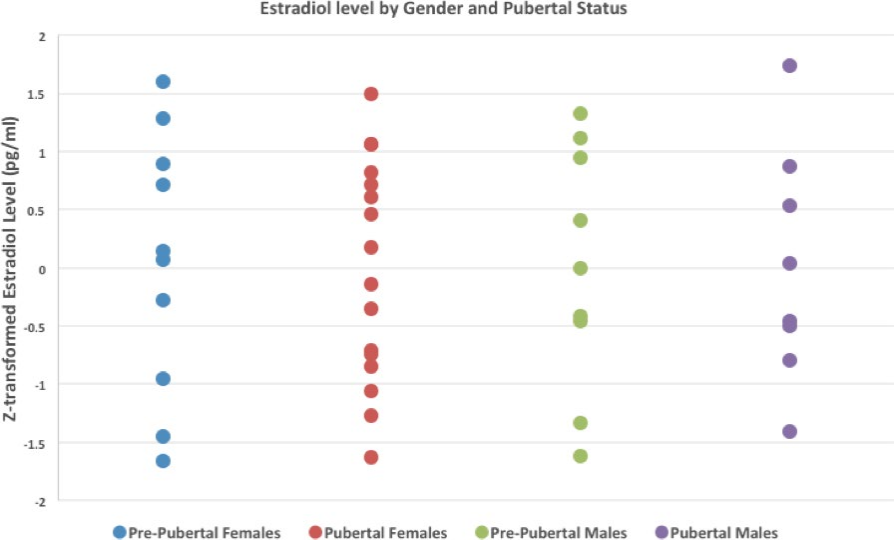
Normalized estradiol values divided in by gender and pre‐ and postpuberty Groups

At each visit during the assessment day, all subjects provided two separate 1‐3cc samples of saliva at two time points between 12 and 6 p.m. The maximum range for the collection of the two hormonal time points was 7 hr and 40 min. Saliva was collected while the subject was relaxed and not after potentially stressful procedures (e.g., MRI). Samples were collected, stored at –20 to −80°C, and shipped in batches from each site to UCLA. Samples were assayed by published RIA methods for estradiol in Dr. McCracken's laboratory at UCLA. Estradiol level was moderately skewed 0.89 (*SE* = 0.34) and kurtosis 0.10 (*SE* = 0.67), so appropriate log10 transformation was performed. Log10‐transformed estradiol levels (skewness −0.05 (*SE* = 0.34) and kurtosis −0.985 (*SE* = 0.67)) were included as regressors in all subsequent multiple regression analyses. An average of the two time points was used for further correlation and regression analyses, controlling for time of collection. Due to the strong hormonal impact on pubertal status, the sample (*N* = 55) was divided into gender‐specific prepubertal groups (14 F, 9 M)‐ and postpubertal groups (16 F, 8 M) (as indicated by the Tanner Stage) and used as a categorical variable to measure the impact of puberty on EF (as indicated by the BRIEF subscales), cortical morphometry, and structural connectivity. We tested the multiple regression slopes (β‐weights) of the pre‐ and postpubertal groups (Figure 1) in the cortical morphometry analyses (including BRIEF subscale‐by‐estradiol interactions). We additionally performed independent samples *t* tests comparing pre‐ and postpubertal groups by BRIEF subscales, estradiol level, and structural connectivity.

### 2.4 Imaging data acquisition

#### 2.4.1 Cortical morphometry

High‐resolution, T1‐weighted images were acquired using a 1.5‐Tesla RI scanner from General Electric (GE) or Siemens Medical Systems (Siemens) (Evans & Brain Development Cooperative, 2006). Imaging data were obtained for each participant on the day of or within a maximum of 28 days of psychometric testing at each visit. GE: SPGR, TR = 22 ms, TE = 10–11 ms, flip angle = 30°, sagittal orientation, FoV = 250 × 250, Matrix = 256 × 256 × 124 − 180 slices, 1–1.5 variable mm slice thickness. Siemens: SPGR, TR = 25 ms, TE = 11 ms, flip angle = 30°, sagittal orientation, FoV = 256 × 256, Matrix = 256 × 256 × 160 − 180, 1 mm slice thickness.

#### 2.4.2 Diffusion tensor imaging (DTI)

Data were acquired at a subset of sites (Boston, Cincinnati, Philadelphia, St. Louis) with a diffusion encoded multislice spin‐echo EPI sequence. To avoid orientation bias, data were acquired on a 3 × 3 × 3 mm matrix covering the entire brain with straight axial slices. GE: diffusion encoded spin‐echo EPI, TR = 3s, TE = minimum full, flip angle = 90°, axial orientation, FoV = 192 if brain 19 cm, Matrix = 64 × 64 × 48; if brain larger than 19 cm FoV = 384 with Matrix = 128 × 128 × 60, four series of six diffusion directions *b* = 1,000. Siemens: diffusion encoded spin‐echo EPI, TR = 3 s, TE = minimum full, flip angle = 90°, axial orientation, FoV = 192 if brain <19 cm with Matrix = 64 × 64 × 48; if brain larger than 19 cm FoV = 384 with Matrix = 128 × 128 × 60, four series of six diffusion directions *b* = 1,000.

### 2.5 Image processing

#### 2.5.1 Surface‐based morphometry (SBM)

Cortical reconstruction and volumetric segmentation was performed with the Freesurfer image analysis suite (v5.6.0), which is documented and freely available for download online (http://surfer.nmr.mgh.harvard.edu/). The technical details of these procedures are described in prior publications (Dale, Fischl, & Sereno, 1999). Briefly, this processing includes motion correction and averaging (Reuter, Rosas, & Fischl, 2010) of volumetric T1‐weighted images, removal of nonbrain tissue using a hybrid watershed/surface deformation procedure (Ségonne et al., 2004), automated Talairach transformation, intensity normalization (Sled, Zijdenbos, & Evans, 1998), tessellation of the gray matter white matter boundary, automated topology correction (Fischl, Liu, & Dale, 2001; Ségonne, Pacheco, & Fischl, 2007), and surface deformation following intensity gradients to optimally place the gray/white and gray/cerebrospinal fluid borders at the location where the greatest shift in intensity defines the transition to the other tissue class (Dale et al., 1999; Dale & Sereno, 1993; Fischl & Dale, 2000). Once the cortical models are complete, a number of deformable procedures were carried out for further data processing and analysis including surface inflation (Fischl, Sereno, & Dale, 1999), registration to a spherical atlas which utilized individual cortical folding patterns to match cortical geometry across subjects (Fischl, Sereno, Tootell, & Dale, 1999), parcellation of the cerebral cortex into units based on gyral and sulcal structure (Desikan et al., 2006; Fischl et al., 2004), and creation of a variety of surface‐based data including maps of cortical volume, surface area (SA), thickness, curvature, sulcal depth, and local gyrification index. The resulting probability maps were input into a general linear model (GLM) evaluating regressions between all vertices and BRIEF subscales, estradiol level, as well as the BRIEF subscale interaction with estradiol level (calculated by multiplying the raw BRIEF score with the estradiol level) controlling for age, gender, time of estradiol collection (when estradiol was present in the analysis), intracranial volume (ICV) and collection site. Vertex‐wise threshold was set at *p* < .001 level. Cluster‐wise threshold was corrected for at *p* < .05 level using nonparametric permutation testing with Monte Carlo simulation.

#### 2.5.2 DTI data

Diffusion tensor imaging images were acquired from the NIHPD database already brain extracted, corrected for eddy current and EPI distortion. DTI images were then further processed using FSL's (v5.0.8, https://fsl.fmrib.ox.ac.uk/fsl/fslwiki/FSL) FDT toolbox (http://fsl.fmrib.ox.ac.uk/fsl/fslwiki/FDT) (Behrens, Johansen‐Berg, et al., 2003; Behrens, Woolrich, et al., 2003). A diffusion tensor model was fit at each voxel, resulting in FA images. FA images from all subjects were registered to an MNI 1 mm skeletonized DTI template using FNIRT, a nonlinear registration tool in FSL. FA values for each subject were then extracted from masks of WM tracts created by the John Hopkins University (JHU) WM atlas: forceps major and minor and bilateral: anterior thalamic radiation, corticospinal tract, cingulum bundle cingulate region (CBc), cingulum bundle hippocampal region (CBh), inferior fronto‐occipital fasciculus (iFOF), inferior longitudinal fasciculus (ILF), superior longitudinal fasciculus (SLF), and uncinate fasciculus. FA values were used in further regression analyses using Pearson correlation coefficient, in SPSS (IBM SPSS Statistics for Macintosh, Version 22.0) using bootstrapping and permutation testing (3,000 simulations) to adjust for small sample size. All analyses were controlled for age, gender, and collection time when estradiol was a included as a variable. Multiple comparison correction was carried out using the Benjamini & Hochberg, 1995 procedure, controlling the false discovery rate (FDR) at *p *< .05. Significant tracts were isolated using the tract visualization program TrackVis (Ruopeng Wang and Van J. Wedeen at Martinos Center for Biomedical Imaging, Massachusetts General Hospital, Charlestown, Mass., USA; https://trackvis.org, Version 0.6.1). A 3 mm‐diameter disk‐shaped ROI was placed in the tract of interest, which allowed for full capture of fibers of interest.

## 3 RESULTS

### 3.1 Behavioral/hormonal results

No significant results were found when investigating the relationship between EF behavior and estradiol level (Table 3A). Given the notable effects of age/gender in the sample, a separate analysis investigating the unique role of age/gender on the relationship between BRIEF subscales and estradiol level was explored (i.e., not controlling for age and gender, Table 3B). This resulted in no significant findings, suggesting age and gender do not have an impact on executive function (EF) behavior and estradiol level in this sample (see Supplementary Material for more detail about these null findings).

**Table 3:** Showing nonsignificant relationship between male and female estradiol level and EF behavior subscales, (A) not corrected and (B) corrected for age/gender, FDR corrected for multiple comparisons

	Male (*N* = 18)		Female (*N* = 30)		Correlation of estradiol with EF Behavior			
	*M*	*SD*	*M*	*SD*	(A) Not Corrected for Age/Gender		(B) Corrected for Age/Gender	
Positive pubertal status	8		17					
Age (years)	13.02	3.02	13.75	3.56	*r*	*p*‐value	*r*	*p*‐value
log‐estradiol level (pg/ml)	0.79	0.24	0.86	0.29	1	[−]	1	[−]
BRIEF behavior regulation	34.29	6.07	36.07	6.79	.03	.86	.03	.96
BRIEF emotional control	12.18	2.19	13.4	3.18	−.05	.74	−.05	.52
BRIEF global executive composite	98.88	17.23	98.87	17.57	.12	.45	.12	.43
BRIEF initiate	11.5	2.5	11.62	2.03	.28	.07	.28	.11
BRIEF inhibition	12.22	3.04	11.93	1.81	.08	.63	.08	.67
BRIEF metacognition	64.22	12.12	62.83	12.91	.18	.24	.18	.23
BRIEF monitor	11.72	2.74	11.66	3.02	.04	.78	.04	.85
BRIEF organization of materials	11.33	2.79	10.69	3.39	.15	.34	.15	.27
BRIEF plan/organize	34.22	23.5	53.59	23.74	.06	.71	.06	1
BRIEF shift	9.72	2.08	10.28	2.15	−.05	.76	−.05	.66
BRIEF working memory	13.22	3.39	12.45	2.71	.16	.31	.16	.27

Abbreviations: BRIEF, Behavior Rating Inventory of Executive Function; EF, executive function; FA, fractional anisotropy; pDLPFC, posterior dorsolateral prefrontal cortex; SA, surface area.

### 3.2 Neuroimaging results

Next, we interrogated the BRIEF subscales, estradiol level, and their interaction, with cortical morphometry and FA. Of note, pubertal status showed no relationship with cortical morphometry or FA. The focus of this paper, therefore, presents *only* estradiol level in relationship to EF, cortical morphometry, and structural connectivity, as well as the impact of BRIEF subscale‐by‐estradiol interaction on brain measurements. The interaction between EF behavior and estradiol level is of particular interest, due to the fact previous studies suggest both variables have a close relationship to cortical morphometry and structural connectivity.

Given the possible effects of age/gender in the sample, an analysis investigating the unique role of age and/or gender on cortical morphometry was explored. The results indicate a negative correlation between age and the right superior frontal gyrus [−log(*p*) = −4.00, *p* = .0001] and right superior temporal sulcus SA [−log(*p*) = −4.00, *p* = .0001], suggesting higher age is related to less SA in these regions. No relationship was observed between gender and cortical morphometry. Because the focus of the paper is concerned with EF behavior and estradiol level, age and gender are used as covariates in the remaining analyses.

**Figure 2:**
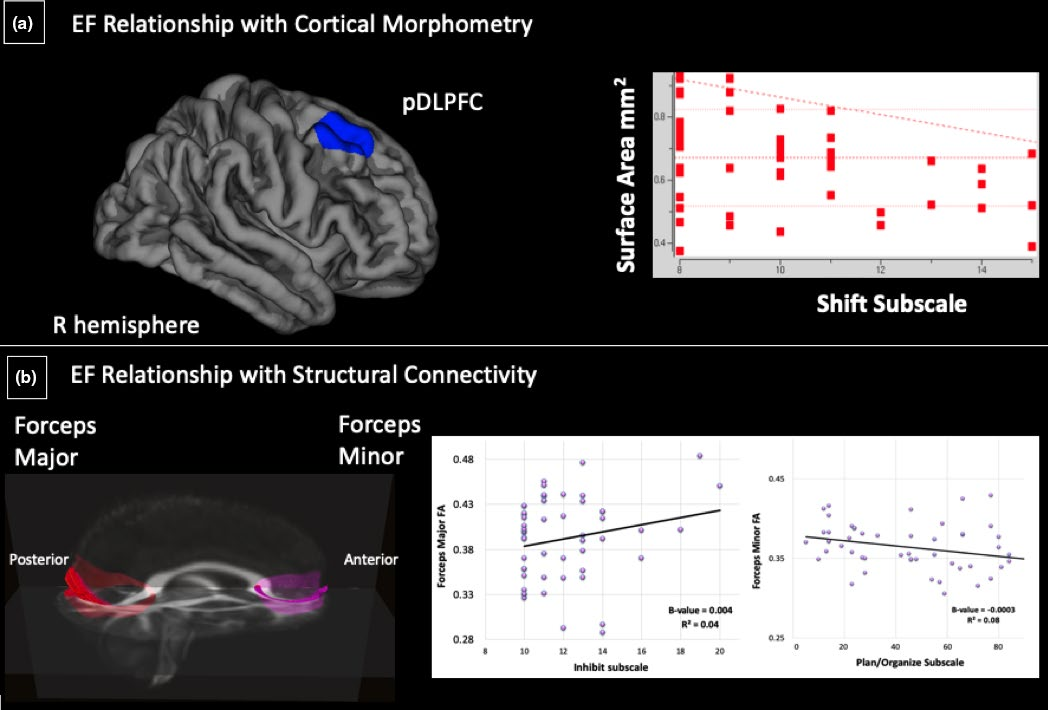
Executive function behavior relationship with cortical morphometry and structural connectivity. (*N* = 51). (a) Significant negative relationship between surface area (SA) in the right pDLPFC and the BRIEF Shift subscale. (b) Significant relationships between two white matter tracts on a template atlas (forceps minor and major) with BRIEF Plan/Organize subscales and BRIEF Inhibit subscales, respectively

**Figure 3:**
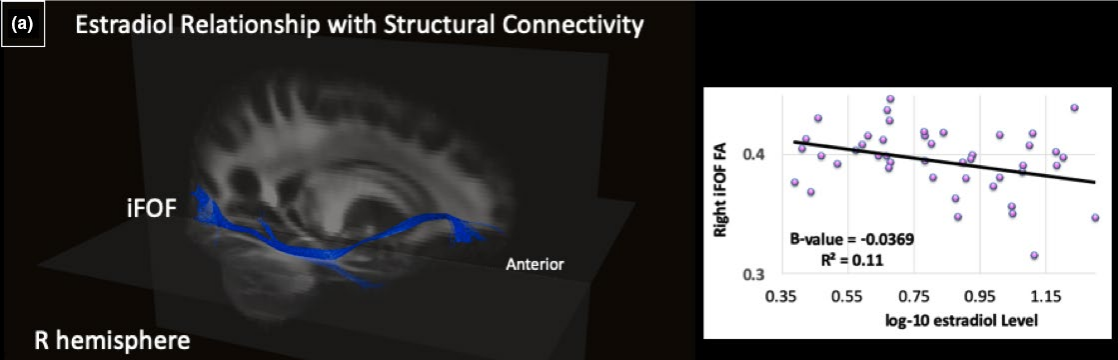
Estradiol relationship with structural connectivity (*N* = 51). (a) Significant negative relationship between right hemisphere iFOF fractional anisotropy (FA) and estradiol level on template atlas

**Figure 4:**
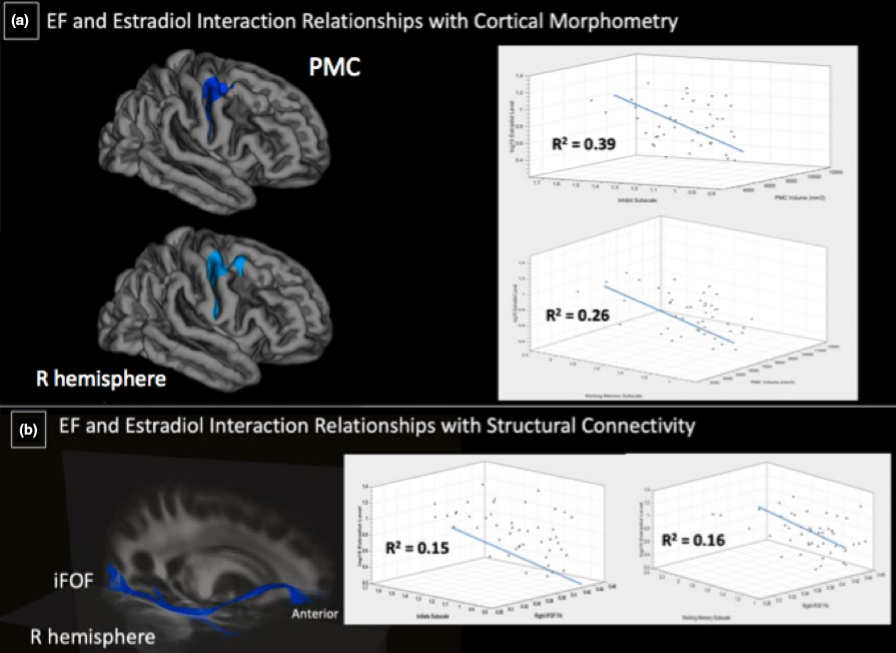
BRIEF subscale and estradiol interaction relationships with cortical morphometry and structural connectivity. (a) Significant negative relationship between volume in the right PMC and the Inhibit‐by‐estradiol interaction and Working Memory‐by‐estradiol interaction. (b) Significant negative relationship between right iFOF FA and Initiate‐by‐estradiol and Working Memory‐by‐estradiol interactions on a template atlas

Therefore, the following sections present morphometric and FA results in the following manner: 3.2.1) relationships of EF with: 3.2.1a) cortical GM and 3.2.1b) FA (Table 4, Figure 2); 3.2.2) relationships of estradiol level with: 3.2.2a) cortical GM and 3.2.2b) FA (Table 5, Figure 3); 3.2.3) relationships of the interaction of EF and estradiol level with: 3.2.3a) cortical GM and 3.2.3b) FA (Table 6, Figure 4). To be consistent throughout the results and discussion, we refer to the positive relationship between EF behavior and estradiol level from the standpoint of higher BRIEF subscale scores (i.e., decreased EF behavior) and elevated estradiol level, to explain their effects on cortical morphometry and FA.

**Table 4:** Relationships of EF behavior with cortical GM and FA

Measure	EF Behavior	Hemisphere	Directionality	Region/Tract
SA	Shift	R	−	pDLPFC
FA	Plan/Organize	None	−	Forceps Minor
FA	Inhibit	None	+	Forceps Major

Abbreviations: EF, executive function; FA, fractional anisotropy; pDLPFC, posterior dorsolateral prefrontal cortex; SA, surface area.

**Table 5:** Relationship of estradiol level with FA

Measure	Variable	Hemisphere	Directionality	Region/Tract
FA	Estradiol	R	−	R iFOF

Abbreviations: FA, fractional anisotropy; iFOF, inferior fronto‐occipital fasciculus.

**Table 6:** Relationships of BRIEF‐by‐estradiol subscales interaction with cortical GM and FA

Measure	BRIEF subscale‐by‐estradiol level	Hemisphere	Direction	Region/Tract
Volume	Inhibit‐by‐estradiol	R	−	PMC
Volume	Working Memory‐by‐estradiol	R	−	PMC
FA	Initiate‐by‐estradiol	R	−	R iFOF
FA	Working Memory‐by‐estradiol	R	−	R iFOF

Abbreviations: BRIEF, Behavior Rating Inventory of Executive Function; iFOF, inferior fronto‐occipital fasciculus; PMC, primary motor cortex.

#### 3.2.1 Executive function results

##### Cortical morphometry

To examine relationships between cortical morphometry and EF (as measured by the BRIEF subscales), each subscale was regressed with surface‐based morphometry (SBM) measures (GMV, surface area, and cortical thickness), controlling for age and gender. This analysis yielded a negative relationship between the Shift subscale and SA in the right pDLPFC [−log(*p*) = −2.19, *p* = .006], indicating decreased ability shifting attention was related to less SA in the right pDLPFC (Table 4, Figure 2).

##### White matter integrity

Having examined gray matter relationships with EF behavior, we next investigated FA. The Plan/Organize subscale showed a negative relationship with FA of the forceps minor (*R* ^2^ = .07, *p* = .01). Conversely, the Inhibit subscale showed a positive relationship with FA in the forceps major (*R* ^2^ = .04, *p* = .02). These results indicated decreased ability putting order into play was associated with lower FA in the forceps minor, while decreased control over impulses was associated with higher FA in the forceps major (Table 4, Figure 2).

#### 3.2.2 Estradiol level results

##### Cortical morphometry

No significant results were observed between cortical morphometry and estradiol level.

##### White matter integrity

Next, we examined the relationship between estradiol level and FA. A negative relationship between estradiol level and FA was observed in the right inferior fronto‐occipital fasciculus (iFOF) (*R* ^2^ = .09, *p* = .01), indicating higher estradiol level related to lower FA in the right iFOF (Table 5, Figure 3).

#### 3.2.3 Executive function and estradiol interaction results

##### Cortical morphometry

Because we hypothesized that estradiol level may interact with EF behavior, we investigated the BRIEF subscale‐by‐estradiol interaction and its effect on cortical morphometry. A negative relationship was observed between the Inhibit‐by‐estradiol interaction and GMV in the right PMC and between the Working Memory‐by‐estradiol and GMV in the right PMC [−log(*p*) = −4.00, *p* = .0001; −log(*p*) = −2.47, *p* = .003, respectively]. These results indicated that increased difficulty inhibiting one's actions and increased levels of estradiol related to less GMV in the right PMC. Additionally, increased difficulty holding information online and increased levels of estradiol related to less GMV in the right PMC (Table 6, Figure 4).

##### White matter integrity

Finally, we examined the relationship between BRIEF subscales‐by‐estradiol and FA interaction. The results showed negative relationships between the Initiate‐by‐estradiol interaction and FA in the right iFOF (*R* ^2^ = .15, *p* = .01), and between the Working Memory‐by‐estradiol interaction and FA in the right iFOF (*R* ^2^ = .16, *p* = .008). The results suggested that increased estradiol level and decreased motivation of task initiation related to lower FA values in the right iFOF (Table 6, Figure 4).

## 4 DISCUSSION

Our results provide comprehensive evidence that individual differences in EF behavior and estradiol level, in a healthy adolescent sample, are linked to variation in aspects of cortical GM morphometry and FA of white matter tracts connecting the cerebral hemispheres and disparate anterior–posterior regions of the brain. Overall, decreased EF behavior related to decreased cortical gray matter morphometry and bidirectional white matter integrity, while increased estradiol level related to decreased white matter tract integrity. Lastly, increased magnitude of the *interaction* between EF behavior and estradiol level related to decreased cortical gray matter morphometry and white matter tract integrity. Below we discuss each finding and its relative implications.

### 4.1 Relationship between executive function and estradiol level

Firstly, we wanted to determine the relationship between estradiol level and EF behavior. We did not find any significant relationships between estradiol level and EF behavior, regardless of correcting or not correcting for age/gender. Previous evidence suggests that estradiol level is indeed related to EF (Hampson & Morley, 2013; Hidalgo‐Lopez & Pletzer, 2017), albeit the results differ depending on the age range of the sample. It is possible our age range (7–18) did not have enough variability to produce statistically significant results.

### 4.2 Relationship between executive function and cortical morphometry

We next aimed to determine how EF behavior, aspects of cortical morphometry, and FA are related. We first hypothesized decreased EF behavior should be associated with decreased aspects of cortical morphometry of the LPFC and decreased FA of tracts that support communication between prefrontal structures, as they have prominent roles in cognitive and emotional function. Our cortical morphometry results demonstrated decreases in EF behavior relating to moving freely from one activity to another, tolerating change, and switching attention (Shift subscale) was associated with decreased SA in the right pDLPFC. Previous research indicates shifting, an EF feature imperative for changing one's own behavior according to environmental contexts, relies on the DLPFC (Karbach & Unger, 2014; Ravizza & Carter, 2008). Furthermore, a recent cortical morphometry study indicates that multitask training leads to increases in right DLPFC GMV (Takeuchi et al., 2014), supporting the notion that shifting behavior depends on the DLPFC.

White matter results indicated decreases in EF behavior relating to putting order into work and play (Plan/Organize subscale) was associated with decreased FA in the forceps minor. Research demonstrates the forceps minor is a fiber bundle which connects the lateral and medial surfaces of the frontal lobes and crosses the midline via the genu of the corpus callosum (Genova, DeLuca, Chiaravalloti, & Wylie, 2013). When damaged by disease, the forceps minor is linked to robustly diminished processing speed and cognitive impairment, indicating its interhemispheric connections between the PFC contribute to EF (Biesbroek et al., 2016; Genova et al., 2013). The association between damage to these tracts and reduced performance in the trail‐making task has been reported in schizophrenia (Perez‐Iglesias et al., 2010) and traumatic brain injury (Kraus et al., 2007). Previous studies in multiple sclerosis (MS) have also noted a correlation between reduced FA in the forceps minor and Paced Auditory Serial Addition Test (PASAT) performance (Van Hecke et al., 2010). Our results therefore echo previous findings: Decreased EF behavior is related with decreased FA of the forceps minor.

Conversely, decreased EF behavior related to controlling impulses (Inhibit subscale) was associated with increased FA in another WM tract, the forceps major. The forceps major is a fiber bundle which connects the occipital lobes and crosses the midline via the splenium of the corpus callosum (Prasad, Upton, Nimgaonkar, & Keshavan, 2015) and is thought to aid visuo‐spatial function (Tamura et al., 2007). Lesions of the forceps major are associated with deficits in multitasking (Burgess, Veitch, de Lacy Costello, & Shallice, 2000), allocation of attentional resources, and other information processing requiring integrated hemispheric function (Rossi et al., 2012). An increase of FA in the forceps major suggests efficient and speedy processing of incoming visuo‐spatial material and thus may result in difficulties inhibiting behavior. Indeed, patients with conditions posited to arise from axonal overconnectivity such as autism spectrum disorder (ASD), attention deficit hyperactivity disorder (ADHD), and schizophrenia exhibit reduced inhibitory control (Solso et al., 2016; Tamm, Barnea‐Goraly, & Reiss, 2012; Taylor, Theberge, Williamson, Densmore, & Neufeld, 2016). Thus, our findings suggest that FA changes in the forceps major affect attention‐based cognitive functions such as impulse control and highlight the complex relationship between white matter structure and EF behavior.

### 4.3 Relationship between executive function, cortical morphometry and structural connectivity

We next investigated whether estradiol level had any relationship to cortical morphometry and FA. We next hypothesized decreased cortical morphometry in the LPFC and reduced FA, as studies indicate that decreased cortical morphometry and FA may both be related to increases in estradiol level in adolescent individuals (Herting et al., 2014, 2012; Peper et al., 2009; Witte et al., 2010). Contrary to our hypothesis, we found no such relationship with cortical morphometry. However, our results indicated that increased estradiol level related to decreased FA of the right iFOF. The iFOF, a long association WM bundle connects the inferior and lateral regions of the PFC through the inferior temporal lobes, terminating in lateral occipital regions (Ashtari, 2012). Research indicates the iFOF plays a critical role in attention and visual processing (Catani & Thiebaut de Schotten, 2008; Wu, Sun, Wang, & Wang, 2016). Our findings mirror results indicating significant differences in long‐range association fibers including the iFOF during adolescence when relationships with estradiol level were considered (Herting et al., 2012). The present study's results point to a relationship between estradiol level and this important WM tract connecting anterior–posterior regions of the cortex which may underlie EF behavior.

### 4.4 Relationship between executive function, cortical morphometry, structural connectivity and estradiol

The final aim of the study was to determine the relationship between the interaction between BRIEF subscales and estradiol (i.e., BRIEF subscale‐by‐estradiol) with cortical GM and FA. At last, we hypothesized that decreased EF behavior would be related to increased levels of estradiol, which may consequently relate to reductions in aspects of cortical morphometry and structural connectivity. Our cortical morphometry findings indicated that decreased EF behavior related to controlling impulses (Inhibit subscale) and holding information online (Working Memory Subscale) coupled with increased estradiol level was associated with less GMV in the PMC. Studies indicate extensive connections exist from the anterior PFC to PMC (Fregni et al., 2005), which are thought to coordinate the integration of higher level EF processes and motor planning in service of goal attainment. Moreover, research demonstrates the LPFC has an increased number of estradiol receptors (Almey, Milner, & Brake, 2015) which may result in increased sensitivity of estradiol in this region posited to underlie EF processes. Indeed, estradiol's impact on working memory is well documented, with high levels of estradiol impairing LPFC‐dependent working memory, while low‐level estradiol weakly facilitating it (Bimonte & Denenberg, 1999; Holmes, Wide, & Galea, 2002; Wide, Hanratty, Ting, & Galea, 2004). Therefore, our results append to existing findings, suggesting that changes in cortical morphometry may reflect more complex interactions between EF behavior and estradiol level affecting the LPFC.

Finally, our study's WM analyses suggested more difficulties with EF behavior related to beginning an activity (Initiate subscale), holding information online when completing a task (Working Memory subscale), and elevated estradiol level were associated with lower FA in the right iFOF. Previous research indicates that elevated estradiol level is related to decreases in EF behavior in adolescents (Lenroot & Giedd, 2010; Peper et al., 2009, 2011) and that FA in the iFOF may be an important neural correlate of EF (Santiago et al., 2015). Thus, our results suggest an important relationship between the interaction of EF behavior and estradiol level on FA in the iFOF, a WM tract providing communication between disparate anterior‐posterior brain regions, putatively underlying EF behavior.

To our knowledge, this is the only study investigating how individual differences in EF behavior and estradiol level relate to aspects of cortical morphometry and FA in a healthy, adolescent population. Our study implies that decreased EF behavior and elevated estradiol level relate to decreased aspects cortical morphometry and FA. Specifically, EF behavior and its interaction with estradiol level related to decreases in aspects of cortical morphometry in the pDLPFC, comprising the LPFC, an area well known to subserve goal‐directed behavior (Asplund, Todd, Snyder, & Marois, 2010; Yamagata, Nakayama, Tanji, & Hoshi, 2012). Further, EF behavior and its interaction with estradiol level were associated with bidirectional differences of FA measurements in interhemispheric connections (forceps minor and major, respectively) and long‐range association fibers (iFOF) connecting anterior–posterior regions of the cortex. Thus, the results imply that variation in EF behavior and estradiol level relate to WM tracts supporting communication between cortical regions.

The study had several limitations. First, the study design was cross‐sectional and not longitudinal, which prevented depiction of individual trajectories, differences in change, and direct estimation of relationships between change across different morphometric measurements. The conclusions from the present study should be replicated in longitudinal studies. Although a longitudinal approach has many merits, because multimodal imaging and hormonal data were only available for a large enough sample during one visit per participant, our study's aims were only possible with a cross‐sectional approach. Second, although pubertal status was taken into account (using the Tanner Stage), menstrual cycle data were not recorded for the female participants, which could further result in fluctuations in estradiol level across the cycle and affect EF behavior. Thirdly, since the BRIEF subscales are highly intercorrelated, discerning their individual impact on brain morphometry is difficult, but speak to their contribution to EF as a whole. Fourthly, no other hormones related to the menstrual cycle were collected. Literature suggests that during the menstrual cycle, both estradiol and progesterone levels fluctuate rapidly and a difference of a few hours can matter dramatically for estradiol levels. Rapidly changing effects of this hormone, coupled with age differences, suggest that these are important factors to keep in mind when researching the effects of cycling in females. Lastly, the measurement of estradiol level from saliva has drawbacks, especially in an adolescent population. Although great care was taken to understand the relationship between estradiol level, EF behavior and aspects of cortical morphometry and structural connectivity, saliva measurements are greatly affected by the use of exogenous hormones such as birth control or transdermal creams (Lewis, McGill, Patton, Patton, & Elder, 2002). Furthermore, results should be interpreted cautiously due to lack of contraceptive and menstrual cycle data.

Future studies should continue to combine EF behavior (such as the BRIEF subscales), estradiol level, and multimodal neuroimaging methods in order to disentangle the function‐estradiol‐structure relationship in this critical neurodevelopmental period in cortical morphometry and structural connectivity thought to underlie EF processes. Specifically, the roles of peptide hormones like oxytocin and vasopressin should be investigated in the neural development of the adolescent brain and its relationship to EF processes.

## CONFLICT OF INTEREST

None declared.

## Data Availability

Data sharing is not applicable to this article as no new data were created or analyzed in this study.
