# Supplementary material for: Cortical morphometry and structural connectivity relate to executive function and estradiol level in healthy adolescents
Source: Brain Behav. 2019 Sep 30;9(12):e01413. doi: 10.1002/brb3.1413 (PMC6908880; doi:10.1002/brb3.1413)
Supplement: Supplementary file 1 [file BRB3-9-e01413-s001.docx]

**Supplemental Material**

**Methods**

*Testosterone*

Testosterone level was moderately skewed 0.62 (SE = 0.34) and kurtosis -0.82 (SE = 0.67), so appropriate log10 transformation was performed. Log10-transformed testosterone levels (skewness -0.18 (SE = 0.34) and kurtosis -0.58 (SE = 0.67)) were included as regressors in all subsequent multiple regression analyses. An average of the two-time points was used for further correlation and regression analyses, controlling for time of collection. In order to better understand the true effect of hormonal level on brain morphology, we conducted an additional analysis whereby log-10 transformed raw testosterone values were normalized (Z scores) within males and females separately, and the normalized values were subsequently used in all cortical morphometry analyses (controlling for pubertal status, collection time, gender, and intracranial volume, corrected for multiple comparisons using FDR, p=0.003). We tested the multiple regression slopes (β-weights) of the pre and post pubertal groups (shown in the Figure 1 below) in the cortical morphometry analyses (including BRIEF subscale-by-testosterone interactions). We additionally performed independent sample T-tests comparing pre and post-pubertal groups by BRIEF subscales, testosterone level and structural connectivity.


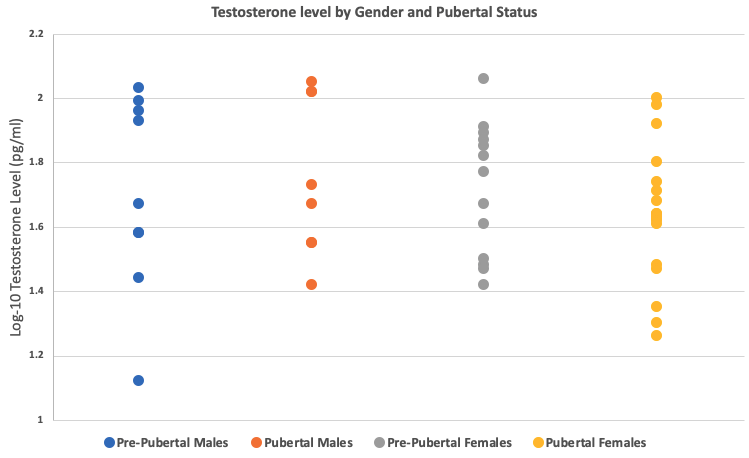


Figure 1: Testosterone level, split by gender and pubertal status as determined by the Tanner Stage. There were no significant differences between any of the groups.

**Results**

*Behavioral/Hormonal Results*

The BRIEF Global Executive Composite score showed a negative relationship with testosterone level (r=-.41, *p*=.003, Table 1A), when age and gender were not controlled for. Given the notable effects of age/gender in the sample, a separate analysis investigating the unique role of age/gender on the relationship between BRIEF subscales and testosterone level was explored (i.e. not controlling for age and gender, Table 1B). This resulted in the previous negative relationship between the BRIEF Global Executive Composite score and testosterone to remain significant (r=-0.46, *p*=.001, Table 1B), with additional negative relationships observed between testosterone level and the BRIEF Metacognition subscale (r=-.49, *p*=.001) and the BRIEF Organization of Materials subscale (r=-0.34, *p*=.003).

|  | **Male**  **(N=18)** | | **Female (N=30)** | | **Correlation of testosterone with EF Behavior** | | | | |
| --- | --- | --- | --- | --- | --- | --- | --- | --- | --- |
|  | M | SD | M | SD |  | ***A.    Not*** Corrected for Age/Gender | | ***B.*** Corrected for Age/Gender | |
| **Positive Pubertal Status** | 8 |  | 17 |  |  |  |  |  |  |
| **Age (years)** | 13.02 | 3.02 | 13.75 | 3.56 |  | r | *p-value* | r | *p-value* |
| ***Log-10 testosterone level (pg/ml)*** | 1.72 | 0.27 | 1.67 | 0.21 |  | 1 | **[-]** | 1 | [-] |
| **BRIEF Behavior Regulation** | 34.47 | 5.91 | 35.16 | 6.81 |  | -.26 | *0.08* | -.26 | *0.07* |
| **BRIEF Emotional Control** | 12.41 | 2.37 | 13.16 | 3.28 |  | -.20 | *0.21* | -.22 | *0.16* |
| **BRIEF Global Executive Composite** | 98.76 | 17.36 | 96.23 | 15.26 |  | **-.41** | ***0.003**** | **-.46** | ***0.002**** |
| **BRIEF Initiate** | 11.71 | 2.41 | 11.48 | 2.06 |  | -.22 | *0.16* | -.25 | *0.11* |
| **BRIEF Inhibition** | 11.76 | 2.41 | 11.67 | 1.67 |  | -.31 | *0.04* | -.33 | *0.04* |
| **BRIEF Metacognition** | 64.29 | 12.49 | 60.73 | 10.51 |  | -.42 | *0.01* | **-.49** | ***0.001**** |
| **BRIEF Monitor** | 11.58 | 2.76 | 11.2 | 2.58 |  | -.29 | *0.07* | -.33 | *0.04* |
| **BRIEF Organization of Materials** | 11.47 | 2.81 | 10.5 | 3.2 |  | -.34 | *0.03* | **-.34** | ***0.003**** |
| **BRIEF Plan/Organize** | 37.13 | 23.34 | 49.43 | 23.98 |  | -.39 | *0.01* | -.14 | *0.62* |
| **BRIEF Shift** | 9.93 | 2.11 | 10 | 2.07 |  | -.16 | *0.32* | -.16 | *0.32* |
| **BRIEF Working Memory** | 13.12 | 3.39 | 12.13 | 2.49 |  | -.26 | *0.09* | -.33 | *0.03* |

Table 1: Relationships between Male and Female testosterone level and EF Behavior subscales, A) ***Not*** Corrected and B) Corrected for age/gender, FDR corrected for multiple comparisons, **p<0.003.**

An independent-samples t-test was conducted to compare testosterone level in pre and post pubertal status grouped by gender. No significant differences were observed between testosterone level in pre pubertal boys (M=1.71, SD=0.20) versus girls (M=1.70, SD=0.30); t(20)=.16, *p*=.21, or between post pubertal boys (M=1.63, SD=0.21) versus girls (M=1.75, SD=0.24); t(24)=-1.2, *p*=.34. We also conducted an additional t-test to assess whether there was a difference in testosterone level between genders as grouped by pubertal status. No significant differences in testosterone level were observed between pre (M=1.71, SD=0.20) and post (M=1.63, SD=0.21); t(29)=-1.05, *p*=.60 pubertal boys or between pre (M=1.70, SD=0.30) and post (M=1.75, SD=0.24); t(15)=.37, *p*=.58 pubertal girls. Since pubertal status as indexed by testosterone level resulted in no significant findings across genders, the findings suggest pubertal status does not have an impact on testosterone level in this sample.

*Neuroimaging Results*

*Testosterone Results*

No significant relationships were observed between any cortical morphometry or white matter integrity as indexed by FA and testosterone level.

*Executive Function and Testosterone Interaction Results*

No significant relationships were observed between BRIEF-by-testosterone interaction and any cortical morphometry or between BRIEF-by-testosterone interaction and white matter tracts as indexed by FA.
